# Challenges and Opportunities for Human Behavior Research in the Coronavirus Disease (COVID-19) Pandemic

**DOI:** 10.3389/fpsyg.2020.01786

**Published:** 2020-07-10

**Authors:** Claudio Gentili, Ioana A. Cristea

**Affiliations:** ^1^Department of General Psychology, University of Padova, Padua, Italy; ^2^Department of Brain and Behavioral Sciences, University of Pavia, Pavia, Italy

**Keywords:** open science, data sharing, social distancing, preprint, preregistration, coronavirus disease, neuroimaging, experimental psychology

The COVID-19 pandemic is a serious public health crisis that is causing major worldwide disruption. So far, the most widely deployed interventions have been non-pharmacological (NPI), such as various forms of social distancing, pervasive use of personal protective equipment (PPE), such as facemasks, shields, or gloves, and hand washing and disinfection of fomites. These measures will very likely continue to be mandated in the medium or even long term until an effective treatment or vaccine is found (Leung et al., 2020). Even beyond that time frame, many of these public health recommendations will have become part of individual lifestyles and hence continue to be observed. Moreover, it is implausible that the disruption caused by COVID-19 will dissipate soon. Analysis of transmission dynamics suggests that the disease could persist into 2025, with prolonged or intermittent social distancing in place until 2022 (Kissler et al., 2020).

Human behavior research will be profoundly impacted beyond the stagnation resulting from the closure of laboratories during government-mandated lockdowns. In this viewpoint article, we argue that disruption provides an important opportunity for accelerating structural reforms already underway to reduce waste in planning, conducting, and reporting research (Cristea and Naudet, 2019). We discuss three aspects relevant to human behavior research: (1) unavoidable, extensive changes in data collection and ensuing untoward consequences; (2) the possibility of shifting research priorities to aspects relevant to the pandemic; (3) recommendations to enhance adaptation to the disruption caused by the pandemic.

Data collection is very unlikely to return to the “old” normal for the foreseeable future. For example, neuroimaging studies usually involve placing participants in the confined space of a magnetic resonance imaging scanner. Studies measuring stress hormones, electroencephalography, or psychophysiology also involve close contact to collect saliva and blood samples or to place electrodes. Behavioral studies often involve interaction with persons who administer tasks or require that various surfaces and materials be touched. One immediate solution would be conducting “socially distant” experiments, for instance, by keeping a safe distance and making participants and research personnel wear PPE. Though data collection in this way would resemble pre-COVID times, it would come with a range of unintended consequences (Table 1). First, it would significantly augment costs in terms of resources, training of personnel, and time spent preparing experiments. For laboratories or researchers with scarce resources, these costs could amount to a drastic reduction in the experiments performed, with an ensuing decrease in publication output, which might further affect the capacity to attract new funding and retain researchers. Secondly, even with the use of PPE, some participants might be reluctant or anxious to expose themselves to close and unnecessary physical interaction. Participants with particular vulnerabilities, like neuroticism, social anxiety, or obsessive-compulsive traits, might find the trade-off between risks, and gains unacceptable. Thirdly, some research topics (e.g., face processing, imitation, emotional expression, dyadic interaction) or study populations (e.g., autistic spectrum, social anxiety, obsessive-compulsive) would become difficult to study with the current experimental paradigms (Table 1). New paradigms can be developed, but they will need to first be assessed for reliability and validated, which will undoubtedly take time. Finally, generalized use of PPE by participants and personnel could alter the “usual” experimental setting, introducing additional biases, similarly to the experimenter effect (Rosenthal, 1976).

**Table 1:** Possible consequences of non-pharmacological interventions for COVID-19 on human behavior research.

	**Facemasks/shields**	**Gloves and disinfection practices**	**Safety distance (e.g., 1 m, 2 m)**
Changes	Facial features	Haptic perception	Social interaction
	Breathing patterns	Pain processing	Subjective experience of the experiment
	Olfaction		
Examples of research topics affected	Autistic spectrum	Proprioception	Emotion processing
	Imitation	Placebo analgesia	Dyadic interaction
	Attachment	Interpersonal relation	Social stress
	Emotion processing	Social touch	
	Face mimicry	Pain	
	Dyadic interaction	Tactile discrimination	
	Social stress		
	Meditation		
	Relaxation		
	Interoceptive exposure		
	Olfactory discrimination		
	Partner selection		
	Olfactive chemo signaling		
	Disgust processing		
Unintended consequences		Fear of contamination and increased anxiety that disinfection was insufficiently performed, particularly with re-usable equipment (e.g., EEG electrodes, earphones, keyboards)	Reluctance and increased anxiety about being in an indoor, confined space (e.g., magnetic resonance imaging scanner)
		Increased anxiety or reluctance to touch items in the experimental setting, particularly food, or drinks (e.g., outcomes like the Taste test)	Increased preparation time, risk of errors due to omissions, or in complex procedures due to reducing presence in the laboratory (e.g., only one experimenter)

Data collection could also adapt by leveraging technology, such as running experiments remotely via available platforms, like for instance Amazon's Mechanical Turk (MTurk), where any task that programmable with standard browser technology can be used (Crump et al., 2013). Templates of already-programmed and easily customizable experimental tasks, such as the Stroop or Balloon Analog Risk Task, are also available on platforms like Pavlovia. Ecological momentary assessment is another feasible option, since it was conceived from the beginning for remote use, with participants logging in to fill in scales or activity journals in a naturalistic environment (Shiffman et al., 2008). Increasingly affordable wearables can be used for collecting physiological data (Javelot et al., 2014). Web-based research was already expanding before the pandemic, and the quality of the data collected in this way is comparable with that of laboratory studies (Germine et al., 2012). Still, there are lingering issues. For instance, for some MTurk experiments, disparities have been evidenced between laboratory and online data collection (Crump et al., 2013). Further clarifications about quality, such as consistency or interpretability (Abdolkhani et al., 2020), are also needed for data collected using wearables.

Beyond updating data collection practices, a significant portion of human behavior research might change course to focus on the effects of the pandemic. For example, the incidence of mental disorders or of negative effects on psychological and physical well-being, particularly across populations of interest (e.g., recovered patients, caregivers, and healthcare workers), are crucial areas of inquiry. Many researchers might feel hard-pressed to not miss out on studying this critical period and embark on hastily planned and conducted studies. Multiplication and fragmentation of efforts are likely, for instance, by conducting highly overlapping surveys in widely accessible and oversampled populations (e.g., university students). Moreover, rushed planning is bound to lead to taking shortcuts and cutting corners in study design and conduct, e.g., skipping pre-registration or even ethical committee approval or using not validated measurement tools, like *ad hoc* surveys. Surveys using non-probability and convenience samples, especially for social and mental health problems, frequently produce biased and misleading findings, particularly for estimates of prevalence (Pierce et al., 2020). A significant portion of human behavior research that re-oriented itself to study the pandemic could result in to a heap of non-reproducible, unreliable, or overlapping findings.

Human behavior studies could also aim to inform the planning and enforcement of public health responses in the pandemic. Behavioral scientists might focus on finding and testing ways to increase adherence to NPIs or to lessen the negative effects of isolation, particularly in vulnerable groups, e.g., the elderly or the chronically ill and their caretakers. Studies could also attempt to elucidate factors that make individuals uncollaborative with recommendations from public health authorities. Though all of these topics are important, important caveats must be considered. Psychology and neuroscience have been affected by a crisis in reproducibility and credibility, with several established findings proving unreliable and even non-reproducible (Button et al., 2013; Open Science Collaboration, 2015). It is crucial to ensure that only robust and reproducible results are applied or even proposed in the context of a serious public health crisis. For instance, the possible influence of psychological factors on susceptibility to infection and potential psychological interventions to address them could be interesting topics. However, the existing literature is marked by inconsistency, heterogeneity, reverse causality, or other biases (Falagas et al., 2010). Even for robust and reproducible findings, translation is doubtful, particularly when these are based on convenience samples or on simplified and largely artificial experimental contexts. For example, the scarcity of medical resources (e.g., N-95 masks, drugs, or ventilators) in a pandemic with its unavoidable ethical conundrum about allocation principles and triage might appeal to moral reasoning researchers. Even assuming, implausibly, that most of the existent research in this area is robust, translation to dramatic real-life situations and highly specialized contexts, such as intensive care, would be difficult and error-prone. Translation might not even be useful, given that comprehensive ethical guidance and decision rules to support medical professionals already exist (Emanuel et al., 2020).

The COVID-19 pandemic and the corresponding global public health response pose significant and lasting difficulties for human behavior research. In many contexts, such as laboratories with limited resources and uncertain funding, challenges will lead to a reduced research output, which might have further domino effects on securing funding and retaining researchers. As a remedy, modifying data collection practices is useful but insufficient. Conversely, adaptation might require the implementation of radical changes—producing *less* research but of higher quality and more utility (Cristea and Naudet, 2019). To this purpose, we advocate for the acceleration and generalization of proposed structural reforms (i.e., “open science”) in how research is planned, conducted, and reported (Munafò et al., 2017; Cristea and Naudet, 2019) and summarize six key recommendations.

First, a definitive move from atomized and fragmented experimental research to large-scale collaboration should be encouraged through incentives from funders and academic institutions alike. In the current status quo, interdisciplinary research has systematically lower odds of being funded (Bromham et al., 2016). Conversely, funders could favor top-down funding on topics of prominent interest and encourage large consortia with international representativity and interdisciplinarity over bottom-up funding for a select number of excellent individual investigators. Second, particularly for research focused on the pandemic, relevant priorities need to be identified before conducting studies. This can be achieved through assessing the concrete needs of the populations targeted (e.g., healthcare workers, families of victims, individuals suffering from isolation, disabilities, pre-existing physical and mental health issues, and the economically vulnerable) and subsequently conducting systematic reviews so as to avoid fragmentation and overlap. To this purpose, journals could require that some reports of primary research also include rapid reviews (Tricco et al., 2015), a simplified form of systematic reviews. For instance, *The Lancet* journals require a “Research in context” box, which needs to be based on a systematic search. Study formats like Registered Reports, in which a study is accepted in principle after peer review of its rationale and methods (Hardwicke and Ioannidis, 2018), are uniquely suited for this change. Third, methodological rigor and reproducibility in design, conduct, analysis, and reporting should move to the forefront of the human behavior research agenda (Cristea and Naudet, 2019). For example, preregistration of studies (Nosek et al., 2019) in a public repository should be widely employed to support transparent reporting. Registered reports (Hardwicke and Ioannidis, 2018) and study protocols are formats that ensure rigorous evaluation of the experimental design and statistical analysis plan *before* commencing data collection, thus making sure shortcuts and methodological shortcomings are eliminated. Fourth, data and code sharing, along with the use of publicly available datasets (e.g., 1000 Functional Connectomes Project, Human Connectome Project), should become the norm. These practices allow the use of already-collected data to be maximized, including in terms of assessing reproducibility, conducting re-analyses using different methods, and exploring new hypotheses on large collections of data (Cristea and Naudet, 2019). Fifth, to reduce publication bias, submission of all unpublished studies, the so-called “file drawer,” should be encouraged and supported. Reporting findings in preprints can aid this desideratum, but stronger incentives are necessary to ensure that preprints also transparently and completely report conducted research. The *Preprint Review* at eLife (Elife, 2020), in which the journal effectively takes into review manuscripts posted on the preprint server BioRxiv, is a promising initiative in this direction. Journals could also create study formats specifically designed for publishing studies that resulted in inconclusive findings, even when caused by procedural issues, e.g., unclear manipulation checks, insufficient stimulus presentation times, or other technical errors. This would both aid transparency and help other researchers better prepare their own experiments. Sixth, peer review of both articles and preprints should be regarded as on par with the production of new research. Platforms like Publons help track reviewing activity, which could be rewarded by funders and academic institutions involved in hiring, promotion, or tenure (Moher et al., 2018). Researchers who manage to publish less during the pandemic could still be compensated for the onerous activity of peer review, to the benefit of the entire community.

Of course, individual researchers cannot implement such sweeping changes on their own, without decisive action from policymakers like funding bodies, academic institutions, and journals. For instance, decisions related to hiring, promotion, or tenure of academics could reward several of the behaviors described, such as complete and transparent publication regardless of the results, availability of data and code, or contributions to peer review (Moher et al., 2018). Academic institutions and funders should acknowledge the slowdown of experimental research during the pandemic and hence accelerate the move toward more “responsible indicators” that would incentivize best publication practices over productivity and citations (Moher et al., 2018). Funders could encourage submissions leveraging existing datasets or developing tools for data re-use, e.g., to track multiple uses of the same dataset. Journals could stimulate data sharing by assigning priority to manuscripts sharing or re-using data and code, like re-analyses, or individual participant data meta-analyses.

## Author Contributions

CG and IC contributed equally to this manuscript in terms of its conceivement and preparation. All authors contributed to the article and approved the submitted version.

## Conflict of Interest

The authors declare that the research was conducted in the absence of any commercial or financial relationships that could be construed as a potential conflict of interest.
